# Integrating RNA-Seq with GWAS reveals novel insights into the molecular mechanism underpinning ketosis in cattle

**DOI:** 10.1186/s12864-020-06909-z

**Published:** 2020-07-17

**Authors:** Ze Yan, Hetian Huang, Ellen Freebern, Daniel J. A. Santos, Dongmei Dai, Jingfang Si, Chong Ma, Jie Cao, Gang Guo, George E. Liu, Li Ma, Lingzhao Fang, Yi Zhang

**Affiliations:** 1 National Engineering Laboratory for Animal Breeding, Key Laboratory of Animal Genetics, Breeding and Reproduction of Ministry of Agriculture and Rural Affairs, College of Animal Science and Technology, China Agricultural University, Beijing, 100193 China; 2 College of Animal Science and Veterinary Medicine, Henan Agricultural University, Zhengzhou, 450002 China; 3 Department of Animal and Avian Sciences, University of Maryland, College Park, MD 20742 USA; 4 Department of Clinical Veterinary Medicine, College of Veterinary Medicine, China Agricultural University, Beijing, 100193 China; 5 Beijing Sunlon Livestock Development Co Ltd., Beijing, 100076 China; 6 Animal Genomics and Improvement Laboratory, Henry A. Wallace Beltsville Agricultural Research Center, Agricultural Research Service, Agricultural Research Service, USDA, Beltsville, MD 20705 USA; 7 MRC Human Genetics Unit at the Institute of Genetics and Molecular Medicine, University of Edinburgh, Edinburgh, EH4 2XU UK

**Keywords:** GWAS, Holstein, Ketosis, RNA-Seq, Phe-WAS, WGCNA

## Abstract

**Background:**

Ketosis is a common metabolic disease during the transition period in dairy cattle, resulting in long-term economic loss to the dairy industry worldwide. While genetic selection of resistance to ketosis has been adopted by many countries, the genetic and biological basis underlying ketosis is poorly understood.

**Results:**

We collected a total of 24 blood samples from 12 Holstein cows, including 4 healthy and 8 ketosis-diagnosed ones, before (2 weeks) and after (5 days) calving, respectively. We then generated RNA-Sequencing (RNA-Seq) data and seven blood biochemical indicators (bio-indicators) from leukocytes and plasma in each of these samples, respectively. By employing a weighted gene co-expression network analysis (WGCNA), we detected that 4 out of 16 gene-modules, which were significantly engaged in lipid metabolism and immune responses, were transcriptionally (FDR < 0.05) correlated with postpartum ketosis and several bio-indicators (e.g., high-density lipoprotein and low-density lipoprotein). By conducting genome-wide association signal (GWAS) enrichment analysis among six common health traits (ketosis, mastitis, displaced abomasum, metritis, hypocalcemia and livability), we found that 4 out of 16 modules were genetically (FDR < 0.05) associated with ketosis, among which three were correlated with postpartum ketosis based on WGCNA. We further identified five candidate genes for ketosis, including *GRINA, MAF1, MAFA, C14H8orf82* and *RECQL4.* Our phenome-wide association analysis (Phe-WAS) demonstrated that human orthologues of these candidate genes were also significantly associated with many metabolic, endocrine, and immune traits in humans. For instance, *MAFA*, which is involved in insulin secretion, glucose response, and transcriptional regulation, showed a significantly higher association with metabolic and endocrine traits compared to other types of traits in humans.

**Conclusions:**

In summary, our study provides novel insights into the molecular mechanism underlying ketosis in cattle, and highlights that an integrative analysis of omics data and cross-species mapping are promising for illustrating the genetic architecture underpinning complex traits.

## Background

The transition period, known as 3 weeks pre- until 3 weeks post-calving, is a critical time for dairy cows since many metabolic and infectious diseases occur due to dramatic physiological challenges faced by cows (e.g., the negative energy balance, NEB) [1]. Ketosis is one of the most important metabolic disorders during transition period. It is often caused due to the severe imbalance between energy demands (e.g., high milk yield) and energy intake. The incidence of ketosis is as high as 15–30% in the dairy industry, and cows with high milk yield predispose to ketosis [2], leading to huge economic losses worldwide. For instance, each case of ketosis costs $ 77.00–180.91 and ¥ 3200 in the U.S. [3] and China [4] Holstein populations, respectively. Ketosis is usually clinically diagnosed by a concentration of β-hydroxybutyrate (BHBA) in plasma greater than 1.4 mmol/L [5–8]. Animals with ketosis are more susceptible to other transition-relevant diseases (e.g., displaced abomasum, DSAB; mastitis, MAST), which together have negative impacts on the performance of production (e.g., reduced milk yield) and reproduction (e.g., infertility) [3, 9].

Ketosis is a complex trait controlled by both genetic and environmental factors, with the estimated heritability ranging from 0.01 to 0.16 [10–13]. Our previous large-scale (*n* ≈ 10 K bulls) genome-wide association study (GWAS) of ketosis (the estimated heritability was 0.012) detected only a few significant loci on *Bos Taurus* autosome (BTA) 14 and BTA16 in Holstein cattle, which together explained a small proportion of its entire genetic variance [10]. This finding strongly suggests a highly polygenetic architecture underlying ketosis. Previous studies proposed that genetic variants of complex traits are enriched in genes with similar biological functions (e.g., Gene Ontology terms) [14–18]. For instance, McCabe et al. (2012) previously demonstrated that differentially expressed genes (DEGs) induced by different energy conditions (i.e., mild NEB and severe NEB) were significantly engaged in fatty acid metabolism and steroid hormone biosynthesis [19]. Therefore, it is of great interest to detect genes that function together during ketosis by using RNA sequencing (RNA-Seq), and then test whether genetic variants of ketosis are enriched in these genes.

**Fig. 1:**
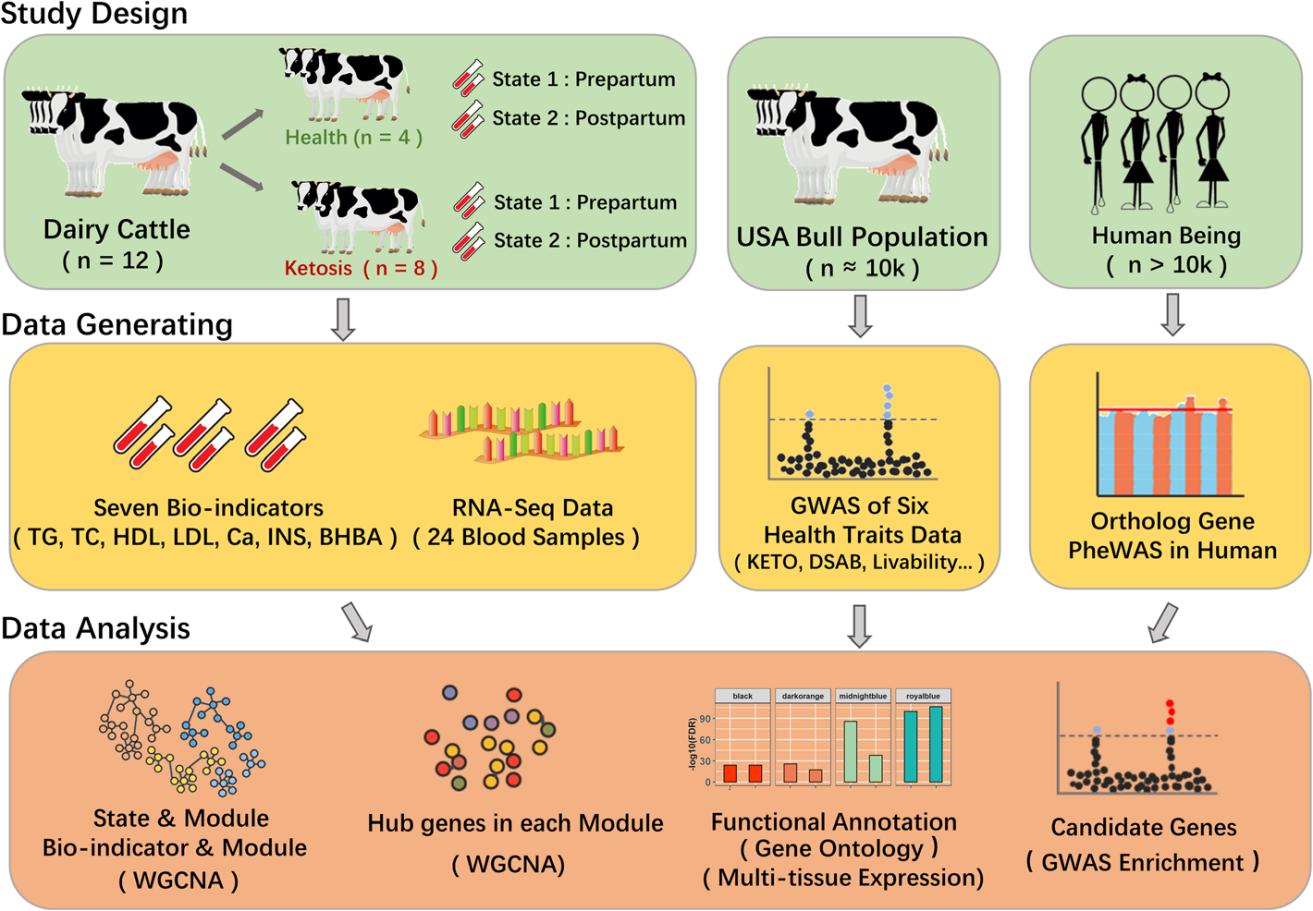
Global framework of the study. The green box (left) represents the experimental design of RNA-Seq study. We selected 12 Holstein cows, among which eight were ketosis (BHBA> 1.4 mmol/L), and the remaining four were healthy (BHBA< 1.4 mmol/L). We collected the whole blood samples from each individual before (2 weeks; prepartum) and after (5 days; postpartum) calving, respectively. The other green boxes (right) demonstrate materials used in genome-wide association studies (GWAS) in cattle and phenome-wide association studies (Phe-WAS) in human. The orange boxes are for data generating, including RNA-Seq and seven blood bio-indicators data from all 24 blood samples, GWAS of six traits (livability; ketosis, KETO; displaced abomasum, DSAB; hypocalcemia, CALC; mastitis, MAST; metritis, METR) and Phe-WAS data (https://atlas.ctglab.nl/). The brown box shows major bioinformatics and statistical analyses involved in the study

In this study (Fig. 1), to explore the genetic architecture underlying ketosis, we generated RNA-Seq of blood leukocytes and biochemical indicators (bio-indicators) of plasma from both healthy and ketosis-diagnosed cows. We then integrated RNA-Seq with large-scale GWAS (*n* ≈ 10 K) of ketosis and other five health traits, including livability, DSAB, hypocalcemia (CALC), MAST and metritis (METR). We further validated our ketosis-candidate genes using the phenome-wide association analysis (Phe-WAS) based on human databases.

## Results

### Summary of RNA-Seq data

In total, we generated 24 RNA-seq data from 12 Holstein cows, including 4 healthy and 8 ketosis-diagnosed ones, before (2 weeks) and after (5 days) calving, respectively. After the quality control of raw RNA-Seq data (in Methods), we obtained a total of 1,286,805,582 clean paired-end reads. By aligning clean data to the cattle reference genome (UMD3.1.1), we obtained an averaged mapping rate of 94.76% (ranging from 93.86 to 95.73%) among all of the 24 samples. We summarized the detailed mapping information for all samples in Additional file 1: Table S1. Ultimately, we observed an average of 13,031 genes (ranging from 12,683 to 13,248) that were expressed (transcripts per kilobase million, TPM > 1) across 24 samples. We then kept 13,600 genes that were expressed in at least one sample and had median absolute deviation (MAD) greater than 0.01 (the top 75% of MAD) for the subsequent analyses.

### Gene co-expression modules associated with ketosis and biochemical indicators

**Fig. 2:**
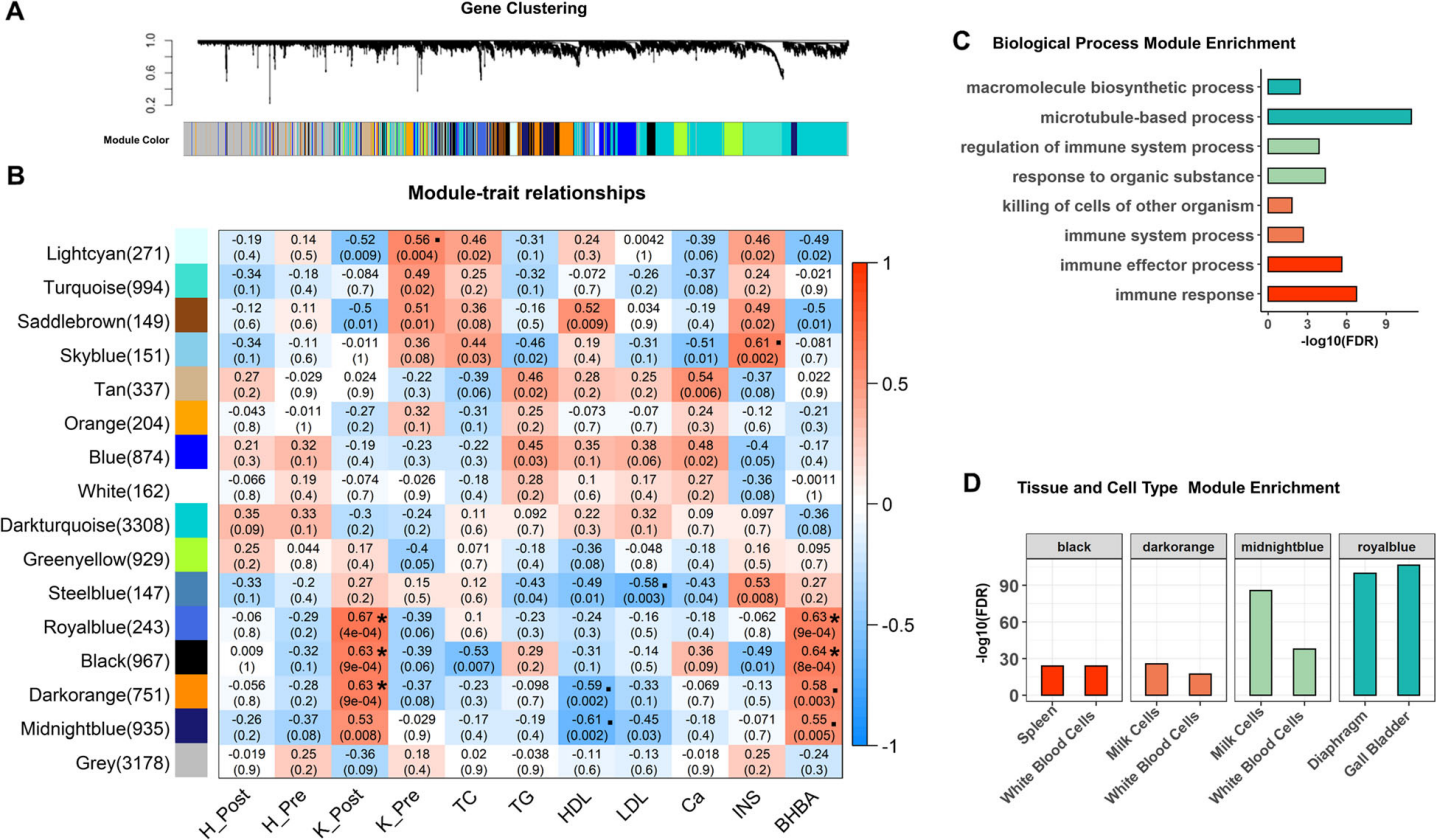
The weighted gene correlation network analysis (WGCNA) for 24 RNA-Seq datasets. **a** 16 gene modules generated from WGCNA analysis. **b** Gene modules associated with four physiological stages (Post-partum Healthy, H_Post; Pre-partum Healthy, H_Pre; Post-partum Ketosis, K_Post; Pre-partum Ketosis, K_Pre) and seven blood bio-indicators (TC: total cholesterol, TG: total triglyceride, HDL: high-density lipoprotein, LDL: low-density lipoprotein, Ca: calcium, INS: insulin, BHBA: beta-hydroxybutyrate). The statistical significance of module-trait relationship is corrected for multiple testing using the FDR method, where “*” and “.” are for FDR < 0.05, < 0.1, respectively. The values in the brackets are the numbers of genes in corresponding modules. **c** The top significantly enriched biological processes for genes in the top four modules associated with the K_Post group. **d** The top significantly enriched tissue/cell types for genes in the top four modules associated with the K_Post group

By employing a weighted correlation network analysis (WGCNA) on all 24 blood leukocytes RNA-Seq data, we detected 16 gene modules (15 co-expression modules and 1 module with the remaining uncorrelated genes), among which the number of genes ranged from 147 to 3178 (Fig. 2a). We then calculated associations of each module with four physiological states (i.e., pre-partum healthy, post-partum healthy, pre-partum ketosis, and post-partum ketosis) and seven blood bio-indicators, including BHBA, total cholesterol (TC), total triglyceride (TG), high-density lipoprotein (HDL), low-density lipoprotein (LDL), calcium (Ca), and insulin (INS) (Additional file 2: Table S2), respectively. Interestingly, we found that three modules, Royalblue, Black, and Darkorange, were significantly (FDR < 0.05) and specifically associated with post-partum ketosis (Fig. 2b). We also found another module, Midnightblue, which tended to be (*P* = 0.008, FDR = 0.10) associated with post-partum ketosis. Gene Ontology enrichment analysis showed that genes in the Royalblue module were significantly (FDR < 0.05) involved in the microtubule-based and macromolecule biosynthetic processes, while genes in the remaining three modules were significantly engaged in immune responses (Fig. 2c, Additional file 3: Table S3). The tissue/cell type-enrichment analysis also confirmed that genes in Royalblue were significantly (FDR < 0.05) enriched for gene with specific expression in digestive and immune systems (e.g., diaphragm and gall bladder), while genes in the remaining three modules were significantly enriched for genes with specific expression in the blood and immune system (Fig. 2d, Additional file 4: Table S4). In addition, we noticed that a module, Lightcyan, appeared to be (FDR < 0.1) associated with pre-partum ketosis. Genes in this module were significantly engaged in the nervous system (Additional file 3: Table S3), which might reflect the cross-talk between the nervous system and digestive/immune systems (i.e., the so-called gut-brain axis) [20–23].

**Fig. 3:**
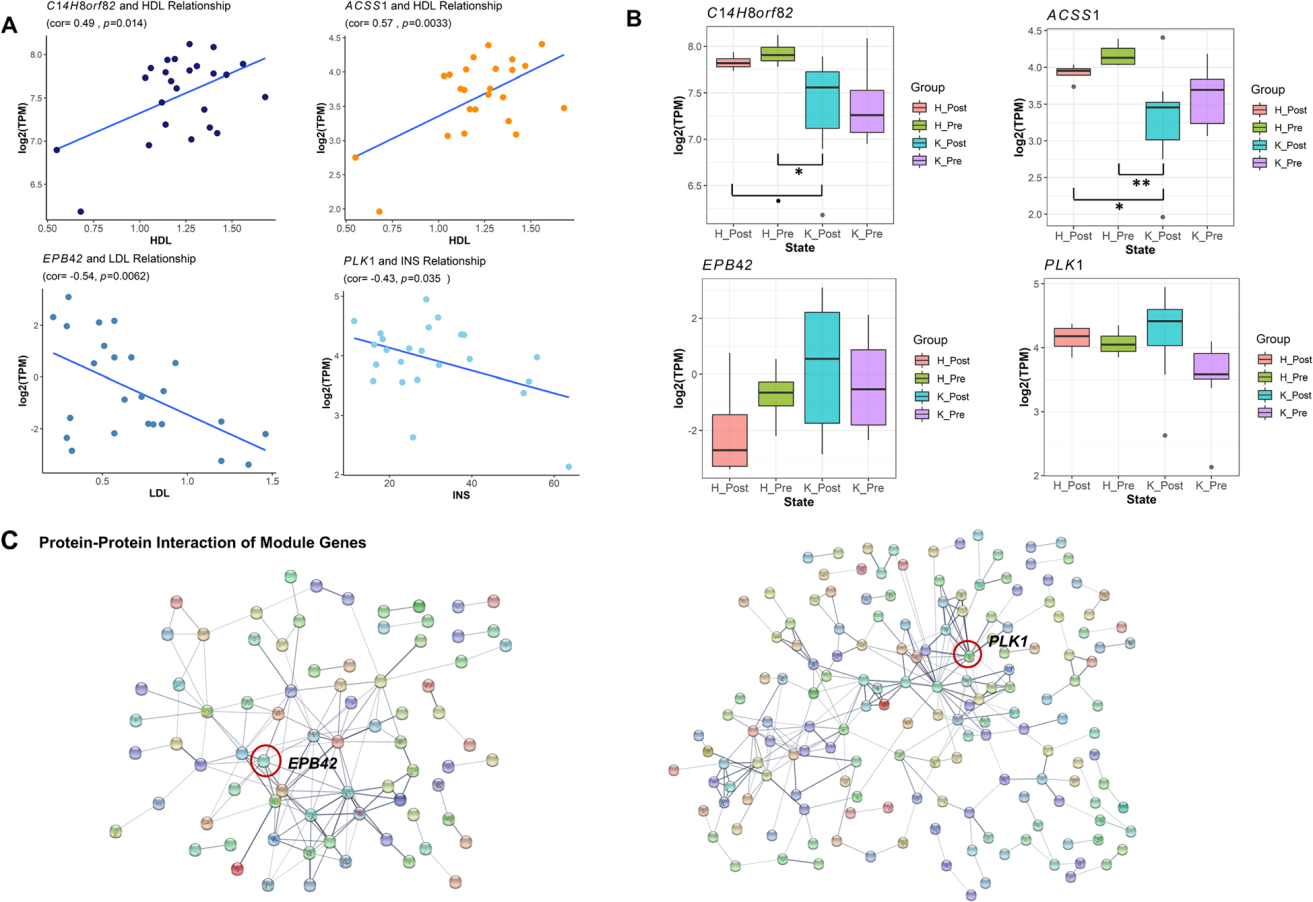
Gene examples in the gene co-expression modules associated with post-partum ketosis and blood biochemical indicators. **a** Scatter plots reflect the correlations between expression levels (log_2_TPM) of genes and levels of blood bio-indicators across 24 blood samples. *C14H8orf82*, *ACSS1*, *EPB2* and *PLK1* belong to Midnightblue, Darkorange, Steelblue and Lightcyan modules, respectively. **b** Boxplots show gene expression levels of four genes among four different physiological stages (Healthy Post-partum, H_Post; Healthy Pre-partum, H_Pre; Ketosis Post-partum, K_Post; Ketosis Pre-partum, K_Pre). The significance level (*P*) is determined by *t*-test. The “**”, “*” and “.” represent *P* less than 0.01, 0.05 and 0.1, respectively. **c** Protein-protein interaction network analysis (STRING v11 database) for genes in Steelblue (left) and Lightcyan (right) modules

We further explored associations of modules with seven plasma bio-indicators (Fig. 2b). As expected, we found that four post-partum ketosis-associated modules were associated with BHBA (FDR < 0.1). We also observed that two modules, Darkorange and Midnightblue, were associated with HDL, while Steelblue and Skyblue modules were associated with LDL and INS, respectively. The pre-partum ketosis-associated module, Lightcyan, tended to be (*P* = 0.02, FDR = 0.13) associated with INS (Fig. 2b). We detected hub-genes in each of these modules (Additional file 5: Table S5). For instance, we found that expression levels of gene *C14H8orf82* (belonging to Midnightblue) and *ACSS1* (Darkorange) were significantly and positively correlated with HDL among 24 samples, while *EPB2* (Steelblue) and *PLK1* (Lightcyan) were significantly and negatively correlated with LDL and INS, respectively (Fig. 3a). Furthermore, we observed distinct expression patterns of these genes in the post-partum ketosis group compared to others (Fig. 3b). For instance, *C14H8orf82* and *ACSS1* had lower expression levels in the post-partum ketosis group than in others, leading to a lower HDL level. In contrast, *EPB2* and *PLK1* exhibited higher expression levels in the post-partum ketosis group, resulting in lower levels of LDL and INS, respectively. The protein-protein interaction analysis also showed that *EPB2* and *PLK1* interacted with many genes within the corresponding modules, indicating their central regulatory roles in these modules (Fig. 3c).

### Gene co-expression modules enriched with GWAS signals of health traits

**Fig. 4:**
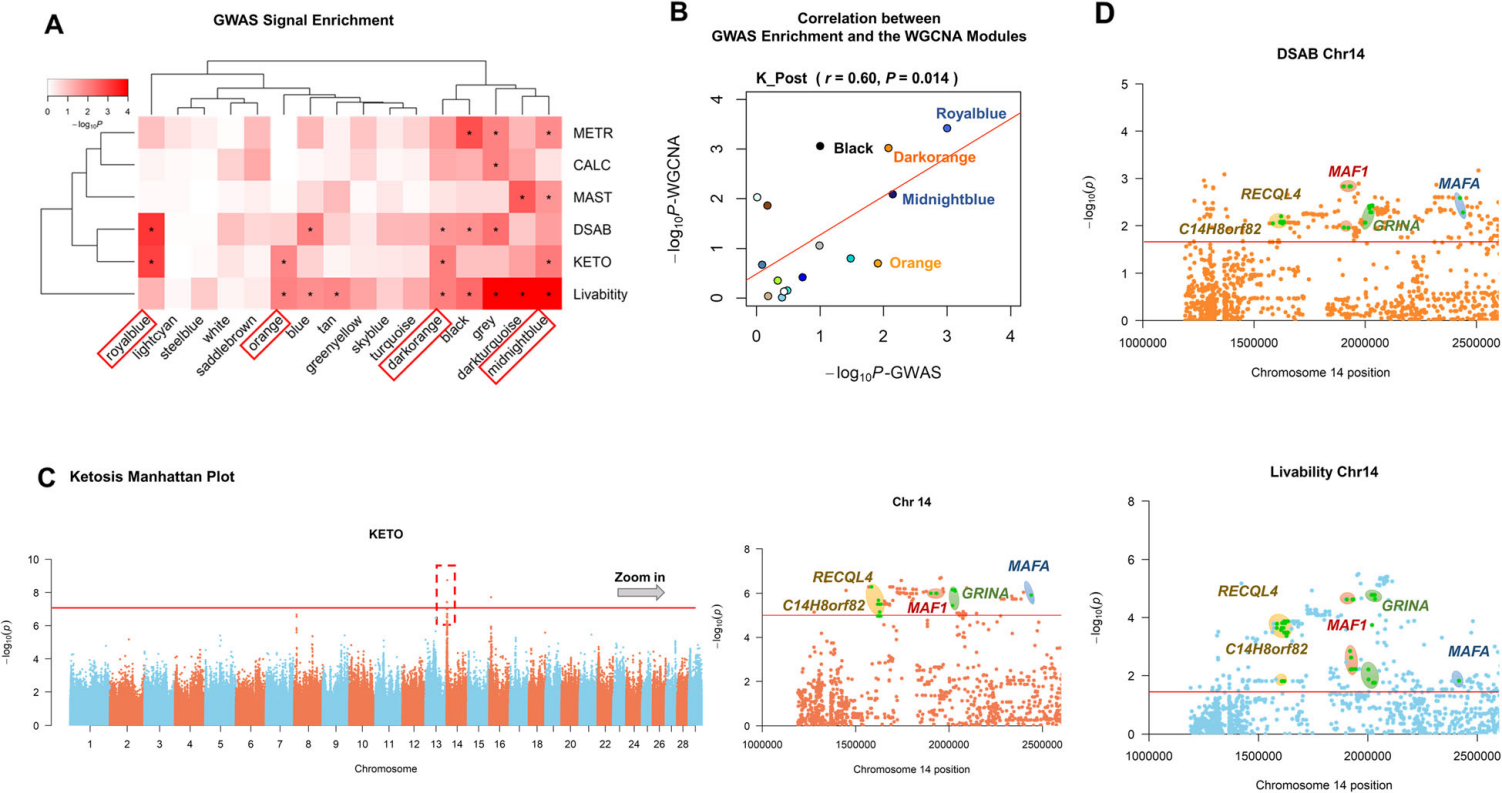
Gene co-expression modules enriched with GWAS signals of ketosis and other five health traits in cattle. **a** GWAS signal enrichment results for all 16 gene modules obtained from WGCNA. The six traits include ketosis (KETO), mastitis (MAST), displaced abomasum (DSAB), metritis (METR), hypocalcemia (CALC) and livability. The statistical significance of enrichment was calculated using the 10,000 times permutation test, followed by multiple testing correction using the FDR method, where “*” means FDR < 0.05. Four modules marked in red are significantly associated with ketosis. **b** Correlation between GWAS enrichment of ketosis and module-states associations from WGCNA across all 16 modules in the ketosis post-partum group, where *r* means Pearson’s correlation and *P* reflects the statistical significance. **c** Manhattan plot for ketosis GWAS (left), where the significant cut-off is *P*-value <5e-08. The red dashed box corresponds to the top QTL of ketosis, which is zoomed in (right) for reflecting locations and significant levels of five candidate genes (red line: *P*-value <10e-05). **d** The locations and significant levels of candidate genes in DSAB and livability (red line: *P*-value < 0.05)

To investigate whether gene co-expression modules were enriched with GWAS signals of ketosis and other health traits, we applied GWAS enrichment analysis for all 16 gene modules across six health traits. As shown in Fig. 4a, several gene modules were significantly (FDR < 0.05) enriched with GWAS signals of these traits, among which ketosis clustered together with DSAB, in line with that both of them are metabolic disorders. We found that four modules, Royalblue, Darkorange, Midnightblue and Orange, were significantly enriched for GWAS signals of ketosis (Fig. 4a). Of note were Royalblue, Darkorange and Midnightblue, whose expression levels were significantly correlated with post-partum ketosis as well (Fig. 2b). By correlating GWAS enrichments of ketosis and module-trait associations from WGCNA across all 16 modules, we only observed a significant correlation (*r* = 0.60, *P* = 0.014) for post-partum ketosis rather than other status (Fig. 4b; Additional file 6: Figure S1). This suggests that transcriptomic alterations induced by post-partum ketosis were biologically and genetically associated with GWAS ketosis. We further detected five candidate genes for ketosis, namely *MAFA*, *C14H8orf82*, *MAF1*, *GRINA* and *RECQL4*, within the four significant modules (Table 1). These genes were located within the top QTL of ketosis on BTA14 (Fig. 4c) [10]. Furthermore, we found that these five candidate genes were also associated (*P* < 0.05) with DSAB and livability (Fig. 4d), providing evidence that they might play polytrophic effects in multiple metabolic disorders.

**Table 1:** Summary of five candidate genes for ketosis

Gene ID	Gene name	Chr.	Position of the top SNP (bp)	SNP effect	*P-*value	Module
ENSBTAG00000000312	*GRINA*	14	2,016,987	0.54	6.94E-07	Midnightblue
ENSBTAG00000012242	*MAF1*	14	1,903,097	0.53	1.00E-06	Midnightblue
ENSBTAG00000026344	*MAFA*	14	2,439,582	0.47	1.21E-06	Midnightblue
ENSBTAG00000046031	*C14H8orf82*	14	1,584,444	0.55	5.17E-07	Midnightblue
ENSBTAG00000010276	*RECQL4*	14	1,621,816	0.51	3.10E-06	Royalblue

### Phenome-wide association analysis (Phe-WAS) for ketosis candidate genes in humans

**Fig. 5:**
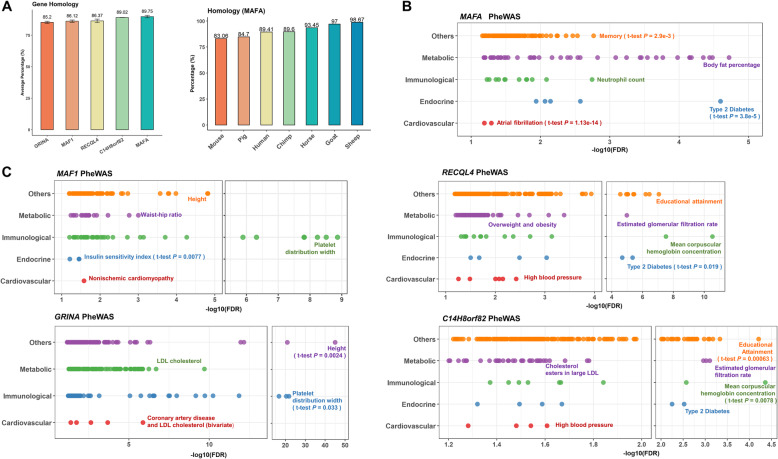
Phenome-wide association analysis (Phe-WAS) for ketosis candidate genes in humans. **a** The bar-plot (left) shows the averaged gene conservation scores of five candidate genes among seven mammalian species. The other bar-plot (right) is for the conservation scores of *MAFA* across seven different mammalian species compared to cattle. **b** Phe-WAS results for *MAFA*, where *P* values are determined by the *t*-test between metabolic traits and the corresponding types of traits. **c** Phe-WAS results for the remaining four candidate genes, where *P* values are calculated by the *t*-test between metabolic traits and the corresponding types of traits

In order to investigate whether candidate genes of cattle ketosis function similarly in humans, we first conducted a homology alignment analysis of these genes. Our results demonstrated that sequences of all five candidate genes were highly conserved (> 80%) among mammals (Fig. 5a left). We took one gene (i.e., MAF BZIP Transcription Factor A - *MAFA*) as an example to show its sequence conservations among seven mammalian species compared with cattle (Fig. 5a right). Then, we conducted Phe-WAS analysis for human orthologues of these candidate genes across 3302 human phenotypes (https://atlas.ctglab.nl/). We found that these genes were significantly associated (FDR < 0.05) with many metabolic traits and other health-relevant traits in humans, such as endocrine and immunological traits, suggesting their conserved roles in the regulation of metabolism and potential pleiotropic effects on many health traits in mammals (Fig. 5b and c; Additional file 7: Table S6). We first took *MAFA* as an example in Fig. 5b. Compared to other types of traits, *MAFA* showed a significantly higher association with metabolic and endocrine traits (e.g., Body fat percentage, FDR = 2.64e-05; Type 2 Diabetes, FDR = 1.9e-03). In addition, we showed Phe-WAS results for the remaining four candidate genes in Fig. 5c, namely *MAF1*, *RECQL4*, *GRINA* and *C14H8orf82*. *MAF1* showed a significantly higher association with immunological traits (e.g., Platelet distribution width, FDR = 1.23e-09) compared to other traits. It was also significantly associated with many endocrine traits (e.g., Insulin sensitivity index, FDR = 0.042; Type 2 Diabetes, FDR = 0.049). *RECQL4* was significantly associated with many endocrine (e.g., Type 2 Diabetes, FDR = 4.53e-06), immunological (e.g., Mean corpuscular hemoglobin concentration, FDR = 2.61e-11) and metabolic traits (e.g., Estimated glomerular filtration rate, FDR = 9.86e-06). It was reported to be associated with nucleic acid binding and annealing helicase activity [24, 25]. *GRINA* showed significant associations with metabolic (e.g., LDL cholesterol metabolism, FDR = 1.83e-07), immunological (e.g., Platelet distribution width, FDR = 1.22e-22) and cardiovascular traits (e.g., Coronary artery disease and low-density lipoprotein cholesterol, FDR = 1.01e-06), and serves to function in apoptotic regulation [26]. *C14H8orf82* was also significantly associated with many metabolic (e.g., Cholesterol esters in large LDL, FDR = 0.032; Estimated glomerular filtration rate, FDR = 7.8e-04), immunological (Mean corpuscular haemoglobin concentration, FDR = 5.83e-05) and endocrine traits (e.g., Type 2 Diabetes, FDR = 0.0041). Our results here demonstrated that ketosis candidate genes detected in cattle might provide novel insights into the molecular mechanism underlying similar complex traits in humans, such as metabolic, immunological and endocrine traits. In turn, our study also demonstrated the potential of cross-species meta-analysis to improve the productivity of the cattle industry.

## Discussion

To our best knowledge, this is the first study to explore the genetic and biological basis of ketosis in dairy cattle by systematically integrating RNA-Seq and large-scale GWAS data. Here, we applied the typical WGCNA strategy - single co-expression network analysis. By using samples of multiple status, a single co-expression network could identify common co-expression modules across status [27]. This analysis strategy has been widely used to detect genes that were associated with developmental stages of diseases, sex and tissues at a system-level [28–31]. For instance, a previous study detected candidate genes for High- and Sub-Fertile reproductive performance in beef cattle using WGCNA [32]. Compared to differential expression analyses at individual gene-level, WGCNA considers the relationship between altering genes as a whole, and reduces the multiple testing burden by focusing on tens of co-expression modules rather than thousands of individual genes. However, it is of note that the status/condition-specific co-expression modules may not be detected in the co-expression networks constructed from samples under multiple conditions, because the correlation signal of the condition-specific modules might be diluted by a lack of correlation in other conditions [27]. To identify modules unique to a specific condition, an alternative strategy, namely differential weighted gene co-expression network analysis (DWGCNA), could be used when sample size is large enough. The DWGCNA approach constructs co-expression networks separately for different datasets to uncover the differences in modules [27, 33, 34].

We validated the detected candidate genes by using cross-species Phe-WAS analysis, which took advantage of rich resources in humans. These results highlight that the integrative analysis of multiple layers of biological data, including cross-species data, is promising to explore the underlying molecular mechanism of complex diseases and traits [15, 18, 35–37]. In this study, we used UMD3.1.1 as reference genome instead of the new assembly (ARS-USD 1.025), as our previous GWAS was conducted based on UMD3.1.1. However, future studies should use the new assembly.

Compared to ketosis, the plasma bio-indicators serve as intermediate phenotypes, which are more directly associated with alterations of gene expression induced by ketosis. The low calcium level in blood can cause ketosis and hypocalcemia, while ketosis leads to insulin resistance, thereby raising the risk of other metabolic diseases [38, 39]. Previous studies proposed that the function of HDL was to transport cholesterol from body tissues to the liver, serving as a “good” lipoprotein [40–42]. This was in line with our findings that the expression of several genes (e.g., *C14H8orf82* and *ACSS1*), which had lower expression levels in the post-partum ketosis group compared to others, were positively correlated with HDL, leading to a lower HDL level in animals with post-partum ketosis (Fig. 3b).

Since gene expression is highly context-specific, it is thus important to choose the “right” tissue at the “right” physiological stages when studying the molecular mechanisms underlying a given trait [18, 43]. For instance, in our study, we observed that gene co-expression modules, which were significantly correlated with post-partum ketosis rather than other status (e.g., pre-partum ketosis), were significantly enriched for GWAS signals of ketosis. This is consistent with findings in our previous study on mastitis, in which we found that the genetic variants of mastitis were specifically and significantly enriched in genes that were differentially expressed in liver at early time points (e.g., 3 h) rather than at the late ones (e.g., 24 h) post *E. coli* infection [18]. It is thus of great interest to collect more RNA-Seq data from multiple time points in the transition period to further explore the causal genes for ketosis in future studies.

In this study, we detected five candidate genes for ketosis, which showed high sequence conservation among mammals. By employing Phe-WAS of their human orthologues on a large range of complex traits and diseases in humans, we confirmed their key roles in metabolism and immunology. For instance, compared to other types of traits, *MAFA* showed significantly higher associations with metabolic and endocrine traits (e.g., Body fat percentage, FDR = 2.64e-05; Type 2 Diabetes, FDR = 1.9e-03). *MAFA* is engaged in insulin secretion, glucose response, and transcriptional regulation [44, 45]. Previous studies reported that *MAFA* was a transcription factor binding RIPE3b, a conserved enhancer element that regulated pancreatic beta cell-specific expression of the insulin gene, which was involved in Insulinomatosis and Diabetes Mellitus [44, 46]. Singh et al. also proposed that *MAFA* was implicated in the regulation of immunomodulatory cytokines such as interferon-β (IFNβ1) [47]. In addition, glucose and lipid metabolic disorders are risk factors that can induce ketosis and immune-relevant diseases like mastitis. Therefore, we considered *MAFA* as a promising candidate gene for ketosis. However, it is required to validate this gene and other four candidate genes in future functional studies.

## Conclusions

In this study, we integrated RNA-seq of blood leukocytes with large-scale GWAS results to detect genes/pathways underlying ketosis in cattle. Our results provide new insights into the molecular mechanism underlying ketosis, and highlights that the integrative analyses of omics data are promising for illustrating the genetic architecture underpinning complex traits and diseases.

## Methods

### Animals and blood samples collection

The experiment was conducted on the dairy farm of Beijing Sunlon Livestock Development Co Ltd. There were 2142 Holstein cows on the farm, of which a total of 78 lactation cows (parity 2–5) entered the dry period at the same time (that is, 2 months before the expected date). Their body condition scores and expected calving dates were similar. These cows were housed in the same pen and fed the same diet. The BHBA concentration in plasma was measured 2 weeks before calving and the fifth day after calving by using Optium ketone test strips with FreeStyle Precision blood ketone meter (Abbott Diabetes Care Ltd., IL, USA). Four cows with BHBA < 1.4 mmol / L on the fifth day after calving were selected as the healthy group; while 8 cows with BHBA > 1.4 mmol / L on the fifth day after calving were considered as ketosis syndrome group. The blood samples were collected from tail (coccygeal) veins of these animals using the following standard procedure. The animal was restrained properly, and its tail was raised vertically until it is horizontal to the ground. The venepuncture area was then disinfected with alcohol. On the midway along the body of a coccygeal vertebra, the needle was inserted perpendicularly to the surface of the skin to a depth of a few millimetres. The blood (10 ml) was then collected using vacutainer tubes. Afterwards, the vacutainer tube was detached from the needle first, and the needle was then removed from the tail vein. The pressure with gauze was applied for 1 minute to ensure adequate hemostasis. The animals used in this study were released after blood sampling. Totally, 24 blood samples (two samples per animal i.e., prepartum and postpartum) were collected, followed by the immediate centrifugation for isolating plasma and leukocytes. Plasma samples were stored at − 20 °C for measuring six blood bio-indicators (e.g., total cholesterol, TC; total triglyceride, TG; high-density lipoprotein, HDL; low-density lipoprotein, LDL; calcium, Ca; and insulin, INS), while leukocytes were stored in the liquid nitrogen for further RNA sequencing. The bio-indicators of blood plasma were determined using commercial assay kits according to the manufacturer’s methods (Laibotairui Technology Development Co Ltd., Beijing, China).

### mRNA extraction and sequencing

We extracted total RNA from 24 blood leukocytes samples using TRIzol reagent (Invitrogen, Carlsbad, CA, USA). Then, we purified the RNA samples (AMPure XP system) and checked their quality using NanoPhotometer® spectrophotometer (IMPLEN, CA, USA). We assessed RNA quality on the Agilent Bioanalyzer 2100 system. The concentration of the RNA samples ranged from 71.8 to 316 ng/μl, and their RNA Integrity Numbers (RIN) were greater than six, which was enough for gene expression analysis [48–50]. We performed the preparation of cDNA library and the RNA sequencing in Novegene Co Ltd. (Beijing, China), generating paired-end reads at 150 bp length (PE150) on the Illumina HiSeq 2500 platform. Ultimately, we obtained an average of 25 million read pairs per sample, which ranged from 21 million to 31 million across 24 samples.

### Bioinformatics analysis of RNA-seq

We conducted quality control for raw reads using FastQC v0.11.3 (http://www.bioinformatics.babraham.ac.uk/projects/fastqc/). We filtered adapters and reads with low quality, in which more than half of bases had quality <= 20 or more than 10% of bases were missing (Ns > 10%), to obtain clean reads. Quality equals to − 10 × log_10_(*e*), where *e* is error rate. We then used Hisat2 v2.0.5 (https://ccb.jhu.edu/software/hisat2/) [51] aligner with default parameters (e.g., −-n-ceil: L, 0, 0.15; −mp: MX = 6, MN = 2; −np: 1; −rdg: 5, 3; −efg: 5, 3; −score-min: L, 0, − 0.2) according to the software manual to map clean reads to the cattle reference genome assembly (UMD3.1.1) [52]. We downloaded the reference genome and all the corresponding annotation files from the Ensembl genome browser 94 (http://oct2018.archive.ensembl.org/Bos_taurus/Info/Index). We used SAMtools v1.6.0 (http://samtools.sourceforge.net/) [53] to transform alignment files from SAM format to sorted BAM format, and then employed the FeatureCounts v1.5.0 (http://bioinf.wehi.edu.au/featureCounts/) [54] to compute read counts for each of the 24,616 annotated Ensembl genes (defined as from transcriptional start site, TSS, to transcriptional end site, TES), including protein-coding genes (*n* = 19,968) and non-coding genes. All software were used with default parameters. For comparing gene expression across genes and samples, we normalized the raw read counts of genes using TPM method [55]. TPM is a normalization method to correct for gene length and sequencing depth to make genes comparable among samples. The raw read count of a gene was first divided by its length in kilobases (RPK), and then all RPK values of genes within a sample were summed up and divided by 1,000,000 to get “per million” scaling factor. The TPM of a gene was obtained through dividing its RPK value by the “per million” scaling factor. We kept genes with TPM (Transcripts Per Million) > 1 and MAD (Median Absolute Deviation, measuring the variability of gene expression across samples) > 0.01 (top 75% of MAD) for the subsequent analyses.

### Weighted gene correlation network analysis (WGCNA)

We employed the WGCNA (v1.12.0), implemented in R, to construct gene co-expression network [56]. Briefly, as recommended by the WGCNA, we used the Variance Stabilizing Transformation (VST) procedure, implemented in DESeq2 [57], to normalize the matrix of read counts, yielding a matrix of values which was approximately homoskedastic (having constant variance along the range of mean values). We transformed this normalized matrix to a similarity matrix based on the pairwise Pearson’s correlation among genes, and then converted the similarity matrix into an adjacency matrix. By using dynamic hybrid cutting method, we clustered genes with similar expression patterns (*r* > 0.9) into 16 distinct modules. We correlated (Pearson’s correlation) eigengenes of 16 modules, defined as the first principle component of the corresponding expression matrix, with physiological states and seven plasma bio-indicators. The hub genes within significant modules were determined with connectivity greater than 30 and gene significance greater than 0.2.

### Single-maker GWAS and GWAS signal enrichment analysis

We obtained summary statistics of single-marker GWAS (*n* ≈ 10,000) for ketosis and five health traits in cattle, which was described previously [10]. Livability as a composite trait is correlated with disease traits, reflecting a cow’s overall ability to stay alive in a milking herd by measuring the percentage of on-farm deaths per lactation [10]. DSAB, highly associated with KETO, occurs when the abomasum fills with gas and moves from the floor to the top of the abdomen. CALC is a metabolic disorder with a low blood calcium level, while MAST is the inflammation of mammary gland. METR is the inflammation of uterus, and often occurs when a cow has a suppressed immune system after calving. Briefly, we considered de-regressed breeding values (predicted transmitting abilities - PTA) of Holstein bulls as phenotypes, while adjusted for all known co-variables, such as herd, year, season, and parity. We used imputed sequence markers (*n* = 2,619,418) with an averaged imputation accuracy of 96.7%, minor allele frequency (MAF) > 0.01 and Hardy-Weinberg Equilibrium (HWE) test (*P* > 10e-06) to conduct GWAS. We employed the following linear mixed model, implemented in MMAP software (https://mmap.github.io/), to test for association of genomic variants with phenotype:

$$ \mathbf{y}=\upmu +\mathbf{X}b+\mathbf{g}+\mathbf{e} $$

where **y** is phenotype, μ is the population mean, **X** is the genotype of a genomic marker (coded as 0, 1, or 2), *b* is the marker effect, **g** ~ $\mathrm{N}\left(\mathbf{0},{\upsigma}_{\mathrm{g}}^2\mathbf{G}\right)$ is the polygenic effect accounting for relationship, while **e** ~ $\mathrm{N}\left(\mathbf{0},{\upsigma}_{\mathrm{e}}^2\mathbf{R}\right)$ is the residual. **G** is the genomic relationship matrix, built using HD markers with MAF > 0.01. **R** is a diagonal matrix with ${\mathrm{R}}_{\mathrm{i}\mathrm{i}}=1/{\mathrm{r}}_{\mathrm{i}}^2-1$, where ${\mathrm{r}}_{\mathrm{i}}^2$ is the reliability of phenotype for the i^th^ individual.

We applied a sum-based method, implemented in the R package for Quantitative Genetic and Genomic analyses (QGG package; http://psoerensen.github.io/qgg/) [58], for GWAS signal enrichment analyses across all 16 gene co-expression modules detected by WGCNA. The sum-based method uses signals of all markers within a pre-defined list of genes (i.e., a gene module), whereas the count-based method only uses signals of significant marker (e.g., *p* < 0.01). Previous studies demonstrated that sum-based approach has equal or better power to detect genomic regions that are enriched with GWAS signals compared to other commonly used methods (e.g., count-based method), particularly in highly polygenic phenotypes [15, 18, 35–37]. Briefly, we calculated the following summary statistics for each gene co-expression module:

$$ T\mathrm{sum}={\sum}_{i=1}^n{b}^2 $$

where *T*_sum_ is the summary statistics for a tested gene module, *b* is the estimate of marker effect obtained in the single-marker GWAS, and *n* is the number of SNPs located in the genes (20 kb up and downstream) within the module being tested. We determined the association of a module with a trait via a 10,000-times circular-genotype permutation test [58]. We calculated an empirical *P* value for the module as the proportion of random *T*_sum_ from permutation greater than the observed *T*_sum_. The details were described previously [36]. In total, we analysed all 16 gene modules across six traits. We corrected raw *P* values for the multiple testing using FDR method implemented in R (*p.adjust* function), and then considered FDR < 0.05 as significant. Within significant modules, we considered genes (including 20 kb up and down-stream of genic regions, i.e., regulatory regions), whose top SNP showed *P* < 10e-05 in GWAS, as candidate genes of ketosis. We used *intersect* function in BEDTools [59] to link SNP to its target gene.

### Other down-stream bioinformatics analyses

To investigate functions of genes in our significant gene co-expression modules, we conducted functional enrichment analysis using the hypergeometric test based on Gene Ontology database and performed protein-protein interaction analysis using STRING v11 database with default settings (https://string-db.org/). We performed tissue and cell enrichment analysis for all 16 gene modules to detect tissues and cell types in which the corresponding genes were more likely to be highly and specifically expressed. Our previous study analyzed 732 RNA-Seq datasets, including 91 different tissue or cell types in cattle, using the same pipeline to detect genes with tissue or cell type-specific expression while accounting for known covariates. The details of the tissue/cell type-specific genes were summarized previously [60] (http://cattlegeneatlas.roslin.ed.ac.uk/). Briefly, for each gene in a given tissue, we computed a *t*-statistics to measure its tissue specificity of expression by fitting a linear regression model (samples of the target tissue were coded as 1, while the remaining ones as − 1). We then ranked genes based on their *t*-statistics (higher value, higher tissue-specificity) in each tissue, and chose the top 5% of genes as tissue-specific genes [43, 60]. We employed the hypergeometric test, similar to the GO enrichment analysis, to detect the enriched tissues and cell types for a given gene module. We adjusted *P* values for multiple testing using the FDR method [61]. We used the BLAST function provided by NCBI (https://blast.ncbi.nlm.nih.gov/Blast.cgi) to obtain the sequence similarities between candidate genes in cattle and their orthologues among other mammalian species. To test whether human orthologues of ketosis-candidate genes are associated with similar traits in humans. We performed the Phe-WAS analysis for human orthologues of these genes across 3302 human phenotypes (https://atlas.ctglab.nl/). Phenome-wide association analysis (Phe-WAS) is a study design that tests associations of a given SNP or gene with a large number of different phenotypes, which is a complementary approach to GWAS. It has been proved useful in recovering previously detected genotype-phenotype associations and in discovering new ones [62, 63].

## Supplementary information

**Additional file 1: Table S1.** Summary of RNA-Seq data mapping information.

**Additional file 2: Table S2.** Summary of seven blood bio-indicators across 24 samples.

**Additional file 3: Table S3.** Gene Ontology (GO) enrichment analysis of five gene modules.

**Additional file 4: Table S4.** Tissue enrichment analysis of all gene modules.

**Additional file 5 Table S5**. Connectivity, significance and membership of genes in modules.

**Additional file 6: Figure S1.** Correlation between GWAS enrichments of ketosis and module-states associations from WGCNA.

**Additional file 7: Table S6.** Phenome-wide analysis results for five candidate genes.

## Data Availability

All 24 high-throughput RNA sequencing data analyzed in this study are deposited in Sequence Read Archive (SRA) database in NCBI under accession number PRJNA605719 (https://www.ncbi.nlm.nih.gov/bioproject/PRJNA605719). The reference genome (ftp://ftp.ensembl.org/pub/release-94/fasta/bos_taurus/dna/Bos_taurus.UMD3.1.dna.toplevel.fa.gz) and gene annotation files (ftp://ftp.ensembl.org/pub/release-94/gtf/bos_taurus/Bos_taurus.UMD3.1.94.gtf.gz) of bovine UMD3.1.1 were downloaded from Ensembl v94 (https://www.ensembl.org/index.html).

## Abbreviations

RNA-Seq: RNA Sequencing
WGCNA: Weighted gene co-expression network analysis
GWAS: Genome wide association studies
Phe-WAS: Phenome-wide association analysis
TC: Total cholesterol
TG: Total triglyceride
HDL: High-density lipoprotein
LDL: Low-density lipoprotein
Ca: Calcium
INS: Insulin
BHBA: Beta-hydroxybutyrate
NEB: Negative energy balance
DMI: Dry material intake
KETO: Ketosis
MAST: Mastitis
DSAB: Displaced abomasum
METR: Metritis
CALC: Hypocalcemia
BTA: *Bos taurus* autosome
MAD: Median absolute deviation
GO: Gene ontology
QTL: Quantitative trait loci
SNP: Single nucleotide polymorphism
RIN: RNA integrity number
FDR: False discovery rate
TPM: Transcripts per kilobase million
TSS: Transcriptional start site
TES: Transcriptional end site
RPK: Reads per kliobase
VST: Variance Stabilizing Transformation
PTA: Predicted transmitting abilities
MAF: Minor allele frequency
HWE: Hardy-Weinberg Equilibrium
DWGCNA: Differential weighted gene co-expressed network analysis
