# Supplementary figures and images for: Integrating RNA-Seq with GWAS reveals novel insights into the molecular mechanism underpinning ketosis in cattle
Source: BMC Genomics. 2020 Jul 17;21:489. doi: 10.1186/s12864-020-06909-z (PMC7367229; doi:10.1186/s12864-020-06909-z)

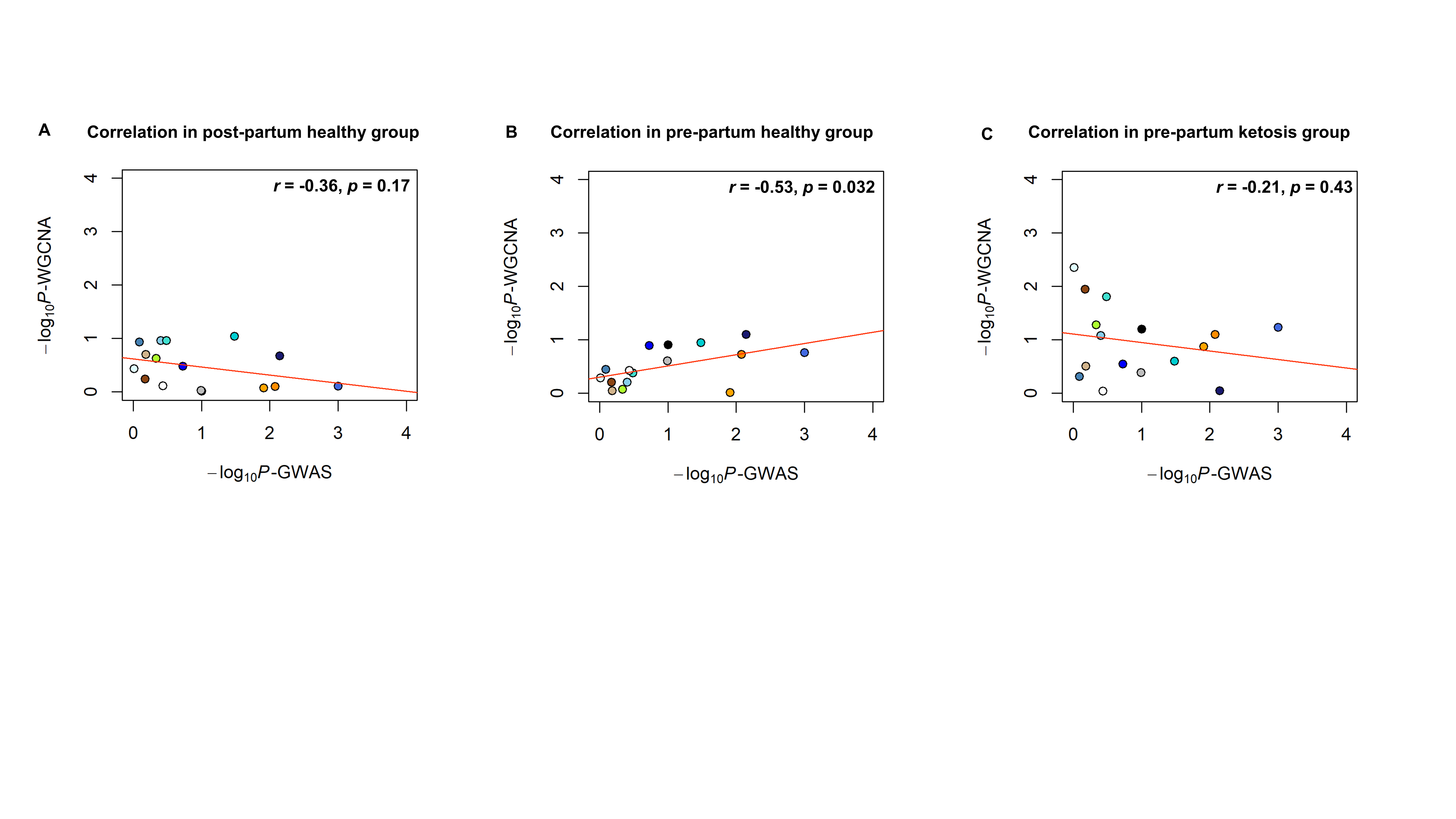

Supplement: Supplementary file 6 — Additional file 6: Figure S1. Correlation between GWAS enrichments of ketosis and module-states associations from WGCNA. [file 12864_2020_6909_MOESM6_ESM.png]
